# TGF‐β1 peptide‐based inhibitor P144 ameliorates renal fibrosis after ischemia–reperfusion injury by modulating alternatively activated macrophages

**DOI:** 10.1111/cpr.13299

**Published:** 2022-06-28

**Authors:** Delun Li, Jian Zhang, Siyu Yuan, Chao Wang, Jiakai Chang, Yan Tong, Ran Liu, Tian Sang, Lili Li, Jijun Li, Qing Ouyang, Xiangmei Chen

**Affiliations:** ^1^ Department of Nephrology, First Medical Center of Chinese PLA General Hospital, Nephrology Institute of the Chinese People's Liberation Army, State Key Laboratory of Kidney Diseases, National Clinical Research Center for Kidney Diseases Beijing Key Laboratory of Kidney Disease Research Beijing China; ^2^ School of Clinical Medicine Guangdong Pharmaceutical University Guangzhou China; ^3^ School of Traditional Chinese Medicine Guangdong Pharmaceutical University Guangzhou China; ^4^ CAS Center for Excellence in Nanoscience, CAS Key Laboratory for Biomedical Effects of Nanomaterials and Nanosafety National Center for Nanoscience and Technology (NCNST) Beijing China

## Abstract

**Objectives:**

Ischemia–reperfusion injury (IRI) is a major cause of chronic renal fibrosis. Currently, numerous therapies have shown a minimal effect on the blockade of fibrosis progression. Here, the therapeutic potential of peptide‐based TGF‐β1 inhibitor P144 in IRI‐induced renal fibrosis and the underlying mechanism were analyzed.

**Materials and Methods:**

The unilateral ischemia–reperfusion injury with the contralateral nephrectomy model was established, and the P144 was administered intravenously 1d/14d after the onset of IRI. The histopathology and immunofluorescence staining were used to detect renal fibrosis and macrophage infiltration. The in vivo fluorescence imaging was used to measure the bio‐distribution of P144. The transwell assays were used to observe the migration of macrophages. RT‐qPCR and western blot were used to analyze TGF‐β1 signaling.

**Results:**

P144 ameliorated the accumulation of extracellular matrix in the kidney and improved the renal function in the unilateral ischemia–reperfusion injury plus contralateral nephrectomy model. Mechanistically, P144 downregulated the TGF‐β1‐Smad3 signaling at both the transcriptional and translational levels and further reduced the TGF‐β1‐dependent infiltration of macrophages to the injured kidney. Additionally, P144 blocked the polarization of macrophages to an M2‐like phenotype induced by TGF‐β1 in vitro, but showed no effect on their proliferation.

**Conclusions:**

Our study showed that the TGF‐β1 peptide‐based inhibitor P144 decreased renal fibrosis through the blockade of the TGF‐β1–Smad3 signaling pathway and the modulation of macrophage polarization, suggesting its potential therapeutic use in IRI‐induced renal fibrosis.

## Graphical abstract

The therapeutic potential of peptide‐based TGF‐β inhibitor P144 in IRI‐induced renal fibrosis and the underlying mechanism were analyzed. P144 downregulated the TGF‐β‐Smad3 signaling at both the transcriptional and translational levels. P144 ameliorated the accumulation of extracellular matrix in the kidney and improved the renal function in the unilateral ischemia–reperfusion injury plus contralateral nephrectomy model. P144 reduced the TGF‐β‐dependent infiltration of macrophages to the injured kidney and the polarization of macrophages to an M2‐like phenotype. Our study suggested the potential therapeutic use of P144 in IRI‐induced renal fibrosis.

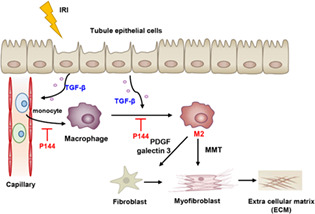

## 1 INTRODUCTION

Acute kidney injury (AKI) is a common clinical syndrome that causes high mortality and influences the long‐term prognosis of patients.^1^ Ischemia–reperfusion injury (IRI) is the major cause of AKI in clinical practice and has been reported to exhibit high prevalence in critical illnesses.^1^ IRI‐AKI is a common cause of chronic kidney disease (CKD) and increases the risk of end‐stage renal disease.^2^ The main pathological manifestation of CKD is renal interstitial fibrosis. Following the persistence or lack of resolution of inflammation after ischemia–reperfusion injury, myofibroblasts are activated by different types of cytokines to produce abundant extracellular matrix, which promotes the progression of renal interstitial fibrosis.^2^

Transforming growth factor‐β (TGF‐β1) is the key regulator of fibrosis in multiple organs, and renal fibrosis has been associated with an increased expression of TGF‐β1.^3^ In particular, TGF‐β1 induces the proliferation, migration, and activation of fibroblasts as well as the deposition of extracellular matrix in the interstitial space.^4^, ^5^, ^6^ Active TGF‐β1 binds to the TGF‐β1 receptor (TGF‐β1R) and exerts biological and pathological functions through the TGF‐β1‐Smad3 signaling pathway in an autocrine and paracrine manner. Inhibition of TGF‐β1 has been shown to attenuate fibrosis in diabetes. However, whether inhibition of TGF‐β1 ameliorates the progression of IRI‐induced renal fibrosis remains to be elucidated.

The TGF‐β1 inhibitory peptide, P144, is a hydrophobic peptide derived from the extracellular ligand‐binding domain of the TGF‐β1 type III receptor.^7^ P144 directly binds to soluble TGF‐β1, thus blocking the binding of TGF‐β1 to the TGF‐β1 type I receptor and the subsequent activation of the signaling pathway.^8^ Previous studies have shown that P144 dampens the progression of liver fibrosis,^9^, ^10^ myocardial fibrosis,^11^ and hypertrophic scars.^12^ However, little is known about the possible use of P144 in the treatment of IRI‐induced renal fibrosis.

In this study, we evaluated the therapeutic potential of P144 in the treatment of IRI‐AKI‐induced renal fibrosis. Our findings revealed that treatment with P144 reduced renal interstitial fibrosis and the expression of fibrotic markers. Interestingly, the protective role of P144 was mediated by the blockade of the M2 polarization of macrophages. These findings suggested the potential use of P144 as an anti‐fibrotic agent in the treatment of renal fibrosis.

## 2 MATERIALS AND METHODS

### 2.1 P144 preparation

Amino acid sequence of P144: TSLDASIWAMMQN. P144 was synthesized by the National Center for Nanoscience and Technology, Beijing, China.

### 2.2 Cell culture

#### 2.2.1 Cell culture and treatment with P144

The RAW264.7 macrophage cell line was purchased from Shanghai Zhongqiao Xinzhou Biotechnology Co., Ltd. (Shanghai, China). RAW264.7 macrophages were cultured in a CO_2_ incubator with Dulbecco's Modified Eagle Medium (DMEM, Corning) supplemented with 10% fetal bovine serum (FCS, Corning). Bone marrow was flushed from femurs and tibias of C57 BL/6J mice, and bone marrow‐derived macrophages (BMDMs) were generated in vitro with macrophage colony‐stimulating factor (M‐CSF, 20 ng/ml, Novoprotein Co., Ltd.) and cultured in DMEM supplemented with 10% FCS. When cells reached a confluence of approximately 80%, they were starved for 24 h and then treated with TGF‐β1 (Novoprotein, 2, 5, 10, and 20 ng/ml) and P144 (200 μg/ml) as indicated.

#### 2.2.2 CCK‐8 assay for cell viability

RAW 264.7 macrophages (1 × 10^4^) were seeded in 96‐well plates and treated with the indicated doses of TGF‐β1 for 24 h. Cell viability was measured using the Cell Counting Kit‐8 (Dongren Chemical Technology Co., Ltd.) following the manufacturer's instructions.

### 2.3 Animals and biochemical measurements

#### 2.3.1 Unilateral ischemia–reperfusion injury (uIRI) and unilateral ischemia–reperfusion injury plus contralateral nephrectomy (uIRIx)

All animal studies were approved by the Animal Ethics Committee of the Chinese PLA General Hospital and Military Medical School, and complied with the ARRIVE guidelines. Male C57 BL/6J mice (Sibefu Biotechnology Co., Ltd.) were used to establish uIRI or uIRIx models. Mice were weighed and anesthetized. Unilateral IRI was performed by clamping the left renal pedicle for 40 min followed by clamp release to allow reperfusion with the contralateral kidney left intact and functional. uIRIx included the removal of the contralateral kidney based on uIRI. Mice were allowed to recover on a 37 °C heating pad with free access to food and water. P144 (1 mg/kg) was administered via the tail vein every other day (D) at D21 postreperfusion for a total of five times. The kidney receiving IRI was harvested for further processing at the indicated time points.

#### 2.3.2 Serum creatinine and urea nitrogen measurement

The levels of serum creatinine and urea nitrogen were detected using the Creatinine Assay Kit (DICT‐500, QuantiChrom. BioAssay Systems) and Urea Assay Kit (DIUR‐500, QuantiChrom. BioAssay Systems) at the indicated times, following the manufacturer's instructions.

### 2.4 Renal tissue staining

#### 2.4.1 Histopathological analysis

Mouse kidneys were fixed in 10% formalin (ACMEC), embedded in paraffin (Beijing Beihua Kangtai Clinical Reagent Co. Ltd), and sectioned to 4 μm thickness. Sections were stained using periodic acid‐Schiff and Masson staining. Images were visualized under an Olympus BX 53 inverted microscope and captured using a color camera.

The area of collagen‐stained area (blue colored area) was measured using ImageJ software (Rasband, W.S., ImageJ, U. S. National Institutes of Health, https://imagej.nih.gov/ij/, 1997–2018.) and calculated from 10 individual high‐power fields in Masson‐stained sections.

#### 2.4.2 Immunofluorescence microscopy

Following fixation with 4% paraformaldehyde (ACMEC) and cryopreservation in 30% sucrose (MACKLIN), the optimal cutting temperature‐embedded kidney tissues were sectioned to 4 μm thickness. Sections were permeabilized with 1% Triton X‐100 (Sigma Aldrich) for 8 min and incubated in a blocking solution (PBS with 1% bovine serum albumin, 0.3% Triton X‐100) for 30 min at room temperature (25°C). Sections were incubated with primary antibodies overnight at 4°C according to the manufacturer's instructions, washed with PBS, and then incubated with the appropriate secondary antibodies in blocking solution for 1 h at room temperature (25°C). After the addition of secondary antibodies, nuclei were stained, and samples were mounted using mounting medium with DAPI (#ab104139; Abcam). All fluorescence images were captured using a confocal FV1000 microscope (Olympus), and images were processed and analyzed using the FV10‐ASW 2.1 Viewer software (Olympus). Primary antibodies included: CD206 (#ab64693; Abcam), F4/80 (#144801, clone BM8; eBioscience), and TGF‐β1 (#ab215715; Abcam).

#### 2.4.3 Flow cytometry

Kidney single‐cell suspension was prepared as described previously. Briefly, the kidney was harvested from mice that were anesthetized. The kidney tissue was cut into 1–2 mm^3^ small pieces with scissors and then digested with 1 mg/ml collagenase I and collagenase IV (#C0130, #C5138, Sigma Aldrich) at 37°C for 30 min. The digested cell solution was passed through a 70 μm cell strainer (Miltenyi Biotec, Bergisch Gladbach) and washed twice with ice‐cold PBS. Flow cytometry was performed using the single‐cell suspension prepared according to the manufacturer's protocols. The reagent and antibodies used were as follows: live‐dead‐7‐AAD (#420403, BioLegend), CD45‐APC‐Cy7 (#157617, BioLegend), F4/80‐FITC (#157309, BioLegend), CD86‐APC (#105011, BioLegend), CD206‐PE (#141705, BioLegend), as well as their isotype controls. FACS data were acquired using a Beckman Coulter cytometer (Cytomics FC 500).

### 2.5 Signaling pathway analysis

#### 2.5.1 Western blotting analysis

Kidney tissues were lysed in radioimmunoprecipitation (Beyotime) assay lysis buffer containing phenylmethylsulfonyl fluoride (Solarbio). Protein concentrations were determined using a bicinchoninic acid assay. Protein samples were separated on a 30% sodium dodecyl sulfate–polyacrylamide gel at 120 V and then transferred to nitrocellulose membranes (Pall Corporation) using a Turbo Transfer System (Bio‐Rad). Membranes were blocked for 1 h at 25 °C and then incubated with the appropriate primary antibodies at 4°C overnight, followed by washing three times with Tris‐buffered saline‐Tween 20 (Sigma Aldrich). Membranes were then incubated with horseradish peroxidase‐conjugated secondary antibodies for 2 h at 25°C and washed with Tris‐buffered saline‐Tween 20. Protein bands were visualized using chemiluminescence with ECL Plus (#P1050; Applygen) according to the manufacturer's instructions. The primary antibodies used in our study were as follows: TGF‐β1 (1:1000, #21898; Proteintech), α‐SMA (1:1000, #55135; Proteintech), Smad3 (1:1000, #ab52903; Abcam), p‐Smad3 (1:1000, ab40854; Abcam), and glyceraldehyde‐3‐phosphate dehydrogenase (GAPDH; 1:1000, #10494‐1‐AP; Proteintech).

#### 2.5.2 RNA expression analysis

RNA from kidney tissues and cells was isolated using the TRIzol method. The mRNA levels of TGF‐β11 and GAPDH were detected by real‐time PCR on the CFX96TM Real‐Time System (Bio‐Rad) using the following primers: TGF‐β11 forward 5′‐CAACAATTCCTGGCGTTACCTTGG‐3′, reverse 5′‐ GAAAGCCCTGTATTCCGTCTCCTT‐3′.

### 2.6 In vivo fluorescence imaging

In vivo fluorescence imaging of P144 was performed as previously described.^13^ Briefly, mice were intravenously injected with 1 mg/kg of Alexa Fluor 647‐conjugated P144 (dye‐to‐protein molar ratio of 4.78:1). Alexa Fluor 647 (Thermo Fisher Scientific) alone served as the control. Mice were anesthetized with isoflurane (Hebei Yipin Pharmaceutical Co. Ltd) and imaged using an IVIS Lumina III imager (PerkinElmer chemagen Technologie GmbH) using identical settings (2‐s exposure, f/stop ¼ 2).

### 2.7 Transwell migration assay

The migration ability of macrophages after treatment with TGF and P144 was evaluated using a transwell migration assay (Corning). Briefly, transwell inserts with 8‐μm pores were pretreated with 1% bovine serum albumin for 1 h. Macrophages were seeded into the inserts at a concentration of 2 × 10^5^ cells per well, while medium containing 10 ng/ml TGF‐β1 with or without P144 was added to the bottom wells. After culturing for 36 h, the inserts were washed with PBS, and macrophages on the upper surface of the insert membrane were scraped off with a cell scraper. Membranes were fixed with methanol (ACMEC) for 20 min and stained with 0.1% crystal violet solution (Solarbio) for 30 min. The number of macrophages that migrated through the membrane pores to the lower surface of the membrane was counted in five fields using an optical microscope.

### 2.8 Statistical analysis

Data are presented as the mean ± standard deviation (mean ± SD). Statistical analysis was performed using *t* test and one‐way ANOVA with GraphPad Prism 8.1. Statistical significance was set at *p* < 0.05.

## 3 RESULTS

### 3.1 Unilateral ischemia–reperfusion injury and unilateral ischemia–reperfusion injury plus contralateral nephrectomy

**FIGURE 1:**
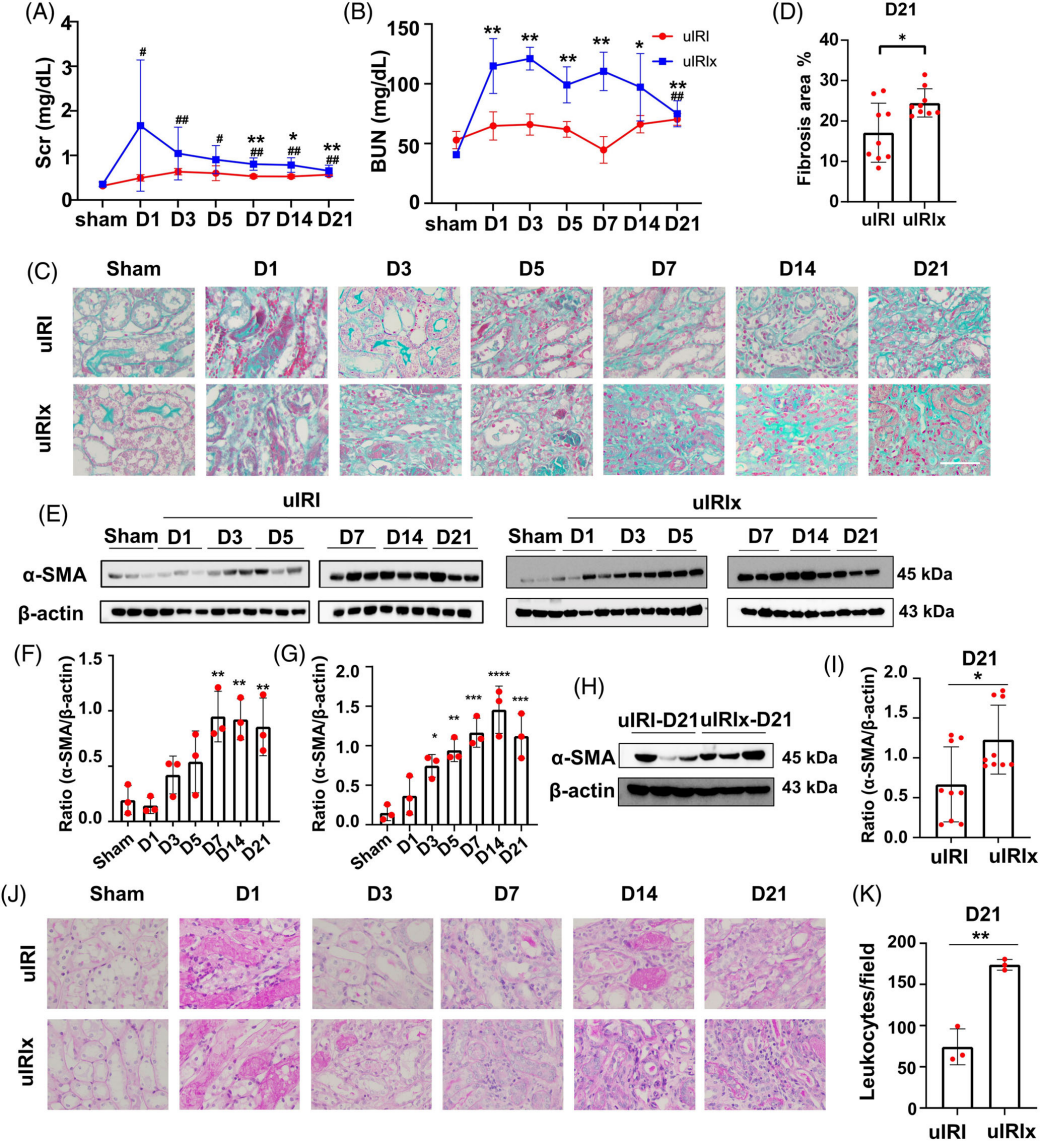
The renal function and pathological characteristics of uIRIx and uIRI models. (A, B) The levels of Scr and Bun in mice in the uIRIx and uIRI models were measured by ELISA. (C, D) Masson staining of uIRIx and uIRI samples at different time points after reperfusion; the percentage of the fibrotic area was quantified. (E–G) Expression of α‐SMA in the kidney after reperfusion detected by western blotting in uIRI and uIRIx mice. (H, I) Expression of α‐SMA in the kidney at D21 after reperfusion detected by western blotting in uIRIx and uIRI mice. (J, K) PAS staining of uIRIx and uIRI samples at different time points after reperfusion; the number of infiltrated leukocytes/field was quantified. Data are presented as the mean ± SD, *n* = 3–9. *p < 0.05, **p < 0.01. Scale bar = 50 μm. α‐SMA, α‐smooth muscle actin; Bun, blood urea nitrogen; PAS, Periodic acid–Schiff; Scr, serum creatinine; uIRI, unilateral ischemia–reperfusion injury model group; uIRIx, unilateral ischemia–reperfusion injury and contralateral nephrectomy

To choose a proper mouse model for the AKI‐CKD transition, we compared the uIRI and uIRIx models. Briefly, for the uIRI model, we induced ischemia–reperfusion injury in the left kidney leaving an intact and functional contralateral kidney, whereas for the uIRIx model, the contralateral kidney was removed. As shown in Figure 1A, the levels of serum creatinine in uIRI mice were increased after reperfusion injury, indicating persistent dysfunction of the ischemic kidney. However, due to the presence of a functional contralateral kidney, we detected only mild changes in the levels of serum urea nitrogen in the uIRI model. In contrast, both the levels of serum creatinine and urea nitrogen were elevated in mice in the UIRIx model after injury. We noticed that these levels were much higher than those in mice in the uIRI model, indicating a more severe renal injury (Figure 1A,B).

To investigate the severity of the renal pathological manifestation, we performed Masson's trichrome staining to evaluate the deposition of extracellular matrix in the interstitial space of the mouse kidney (Figure 1C,D). We found that mice in both the uIRI and uIRIx models demonstrated gradually increasing renal fibrosis after reperfusion injury, with uIRI mice exhibiting greater collagen deposition in the chronic phase of CKD (Figure 1C,D). Consistent with these results, we detected higher levels of expression of the α‐SMA fibrotic marker in the kidney of uIRIx mice compared with that in uIRI mice at D21 (Figure 1I,J), although the level of expression was increased in both models after reperfusion injury (Figure 1E–H). We further observed that besides the production of extracellular matrix in the interstitial space of the kidney, the infiltration of leukocytes also indicated the severity of renal injury and fibrosis. As shown by periodic acid‐Schiff staining (Figure 1K,L), much more leukocytes infiltrated around the fibrotic area in the renal interstitial space of uIRIx mice than in the other model, suggesting a sustained chronic inflammation. Altogether, the uIRIx model better demonstrated kidney pathological impairment and renal function deterioration during the AKI‐CKD transition.

### 3.2 Pattern of TGF‐β1 expression and macrophage infiltration in the IR‐induced fibrotic kidney

**FIGURE 2:**
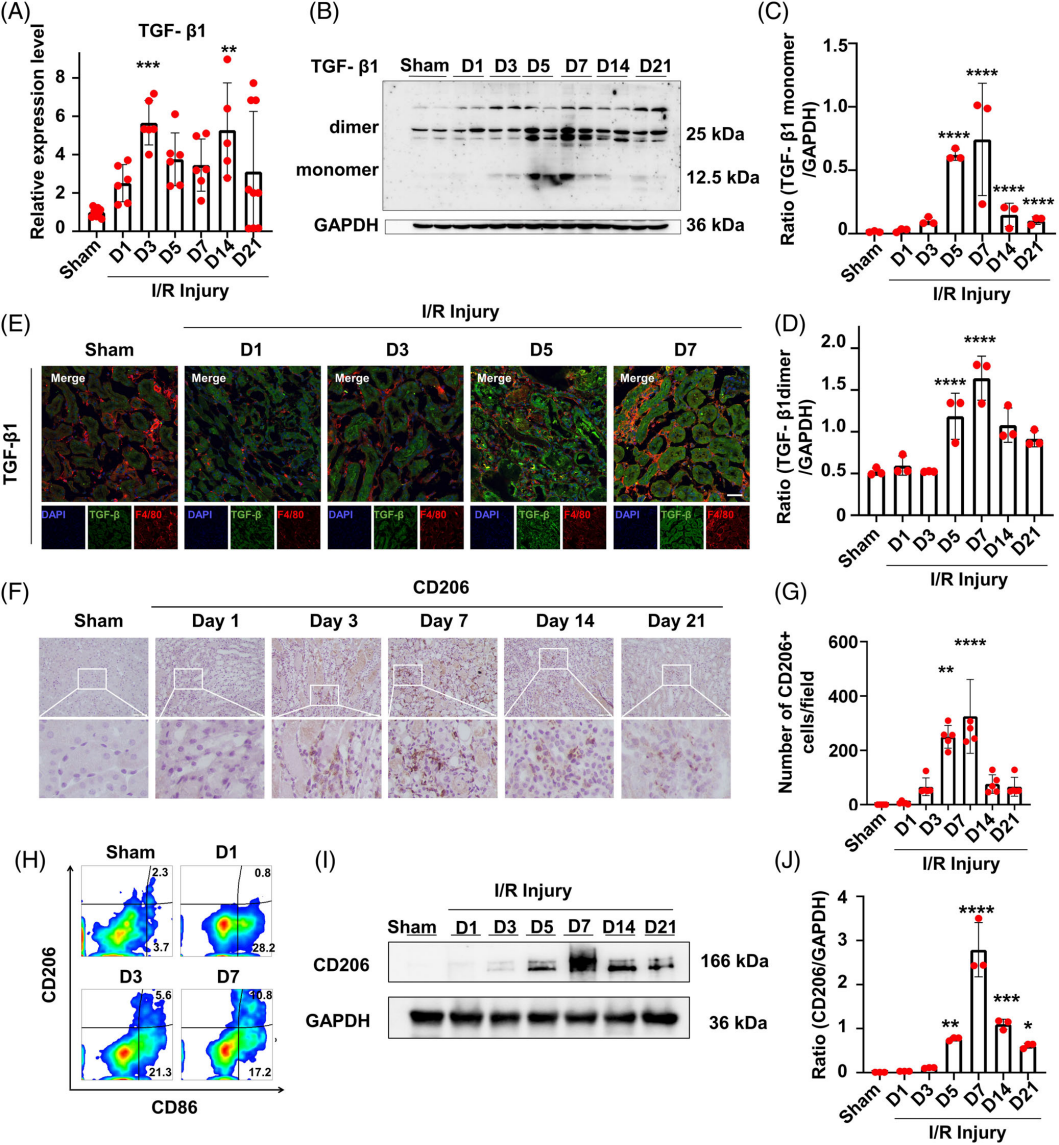
Expression pattern of TGF‐β1 and infiltration of macrophages in the kidney of uIRIx mice. (A) mRNA expression of TGF‐β1 in uIRIx mice measured by RT‐qPCR. (B–D) Expression of TGF‐β1 dimers and TGF‐β1 monomers in uIRIx mice at D1, D3, D5, D7, D14, and D21 after reperfusion detected by western blotting. (E) Expression of F4/80 and TGF‐β1 in uIRIx mice at D1, D3, D5, and D7 after reperfusion detected by immunofluorescence. (F,G) Expression of CD206 in the kidney of uIRIx mice detected by immunohistochemical staining. (H) Expression of CD86 and CD206 in the kidney of uIRIx mice detected by flow cytometry. (I, J) Expression of CD206 in the kidney of uIRIx mice detected by western blotting in uIRIx mice. Data are presented as the mean ± SD, *n* = 3–9. *p < 0.05, **p < 0.01, ***p < 0.001, ****p < 0.0001. Scale bar = 50 μm. TGF‐β1, transforming growth factor‐β; uIRIx, unilateral ischemia–reperfusion injury and contralateral nephrectomy

Among the many growth factors, TGF‐β1 is known to be a key regulator of signaling pathways that promote renal fibrosis, especially in diabetic kidney disease. To further investigate the expression pattern of TGF‐β1 in IRI‐induced fibrosis, we detected the expression of TGF‐β1 at both the transcriptional and translational levels in the kidney of uIRIx mice. We found that the expression of TGF‐β1 mRNA was upregulated during the acute inflammation phase and was then sustained in the late stage of renal fibrosis (Figure 2A). More specifically, we detected that the expression of intact TGF‐β1 was moderately increased during the first 5 d after injury, peaked at D7, and then lasted until D21 (Figure 2B,C). Consistently, we observed that activated TGF‐β1 accumulated in the kidney from D5 to D7 (Figure 2B,D). Thus, we analyzed the localization of the expression of TGF‐β1 using confocal microscopy. As shown in Figure 2E, TGF‐β1 staining was predominantly localized in the renal tubular area rather than in the interstitial space, indicating renal tubular epithelial cells as the major source of TGF‐β1.

Macrophages are the predominant innate immune cells that infiltrate the kidney and participate in inflammation after various host insults. Here, we confirmed the involvement of macrophages in IRI‐induced renal fibrosis using immunofluorescence staining of F4/80 cells. We found that macrophages were mainly localized at the cortex‐medullary junction of the kidney and their number was increased since D3 after IR injury (Figure 2F,G). We also profiled the immune phenotype of macrophages using flow cytometry. We accordingly observed that the number of CD86^+^ M1 macrophages was dramatically increased at D1 and then decreased at D3, whereas the number of CD206^+^ M2 macrophages began to increase at D3 and maintained at a high level at D7 (Figure 2H). In support of these results, western blotting showed that CD206 was significantly increased after renal ischemia–reperfusion from 72 h to 21 days (Figure 2I,J). These findings indicated that macrophages were involved in renal fibrosis induced by ischemia–reperfusion injury, with M2 macrophages being the predominant immune cells at the stage of chronic inflammation and fibrosis.

### 3.3 P144 ameliorated the IRI‐induced renal fibrosis

**FIGURE 3:**
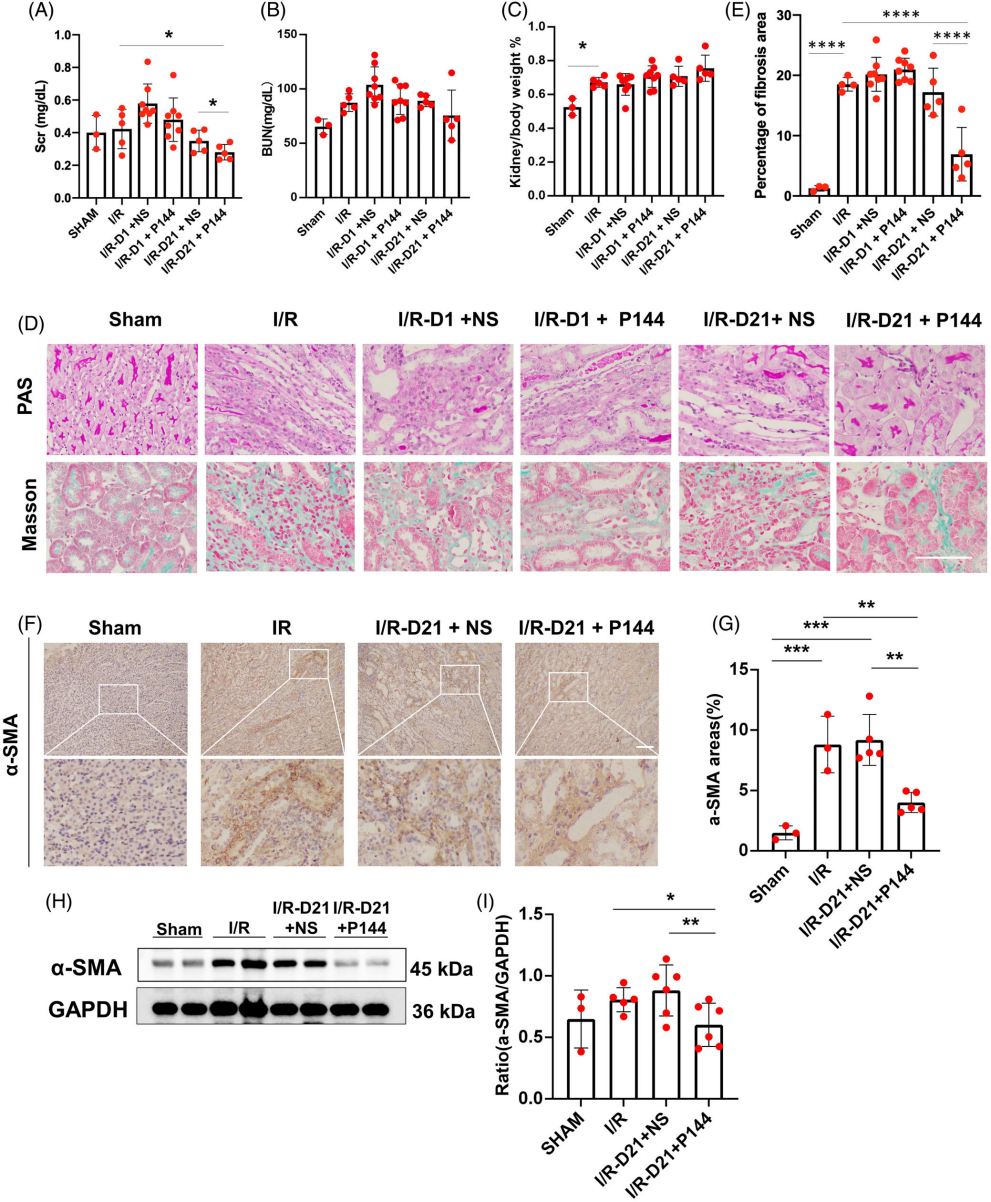
The antifibrotic effect of P144 in IRI‐induced renal fibrosis in vivo. (A, B) The levels of Scr and Bun in uIRIx mice treated with P144 were measured by ELISA. (C) The ratio of kidney/body weight in uIRIx mice treated with P144. (D) PAS staining and Masson staining of kidneys of uIRIx mice treated with P144. (E) The percentage of the fibrotic area was quantified. (F, G) Immunohistochemical staining of α‐SMA in the kidneys of uIRIx mice treated with P144 at D21 after reperfusion. (H, I) Expression of α‐SMA in the kidneys of uIRIx mice treated with P144 at D21 after reperfusion detected by western blotting. Data are presented as the mean ± SD, *n* = 3–8.*p < 0.05, **p < 0.01, ***p < 0.001, ****p < 0.0001. Scale bar = 50 μm. Bun, blood urea nitrogen; IRI, ischemia–reperfusion injury; Scr, serum creatinine; TGF‐β1, transforming growth factor‐β; uIRIx, unilateral ischemia–reperfusion injury and contralateral nephrectomy

To investigate whether treatment with P144 alleviated the progression of renal fibrosis induced by IR injury, and to determine whether the time to start treatment would influence the outcome, we iv injected uIRIx mice with P144. Briefly, mice treated with P144 since D1 post‐IR injury were defined as the early phase treatment group, whereas mice treated since D21 post‐IR injury were defined as the late‐phase treatment group. Mice in both groups were treated every other day for a total of five times. At D35, we collected peripheral blood samples from treated mice to test their renal function, while their IR kidneys were harvested and processed for histological and immunohistochemistry staining. We did not detect any difference in the levels of serum BUN and creatinine between the two different groups receiving the early‐phase treatment, however, the serum creatinine of the late‐phase P144 treatment group was reduced compared with the untreated group and the NS treated group (Figure 3A,B). Moreover, the treatment did not affect the kidney weight (Figure 3C). We did not observe any difference in the renal fibrosis score between mice receiving the early phase treatment and untreated mice; however, we noticed that the renal fibrosis score was significantly reduced in the late‐phase treatment group compared with that in either untreated mice or mice in the early phase treatment group as indicated by periodic acid‐Schiff and Masson's trichrome staining (Figure 3D,E).

To further validate the therapeutic efficacy of P144, we performed α‐SMA staining to investigate whether renal fibrosis was attenuated. We found that at Day 35 post‐IRI, renal fibrosis was alleviated in the presence of P144 (late‐phase treatment), compared with the NS group (Figure 3F,G). In support of these data, we detected that the expression of α‐SMA in the kidney was obviously reduced in the P144 group as indicated by western blotting (Figure 3H–I). These data further substantiated the anti‐fibrotic effects of P144 in the kidney, attenuating the progression of AKI to the chronic stages of the disease.

### 3.4 P144 was enriched in the kidney of uIRIx mice

**FIGURE 4:**
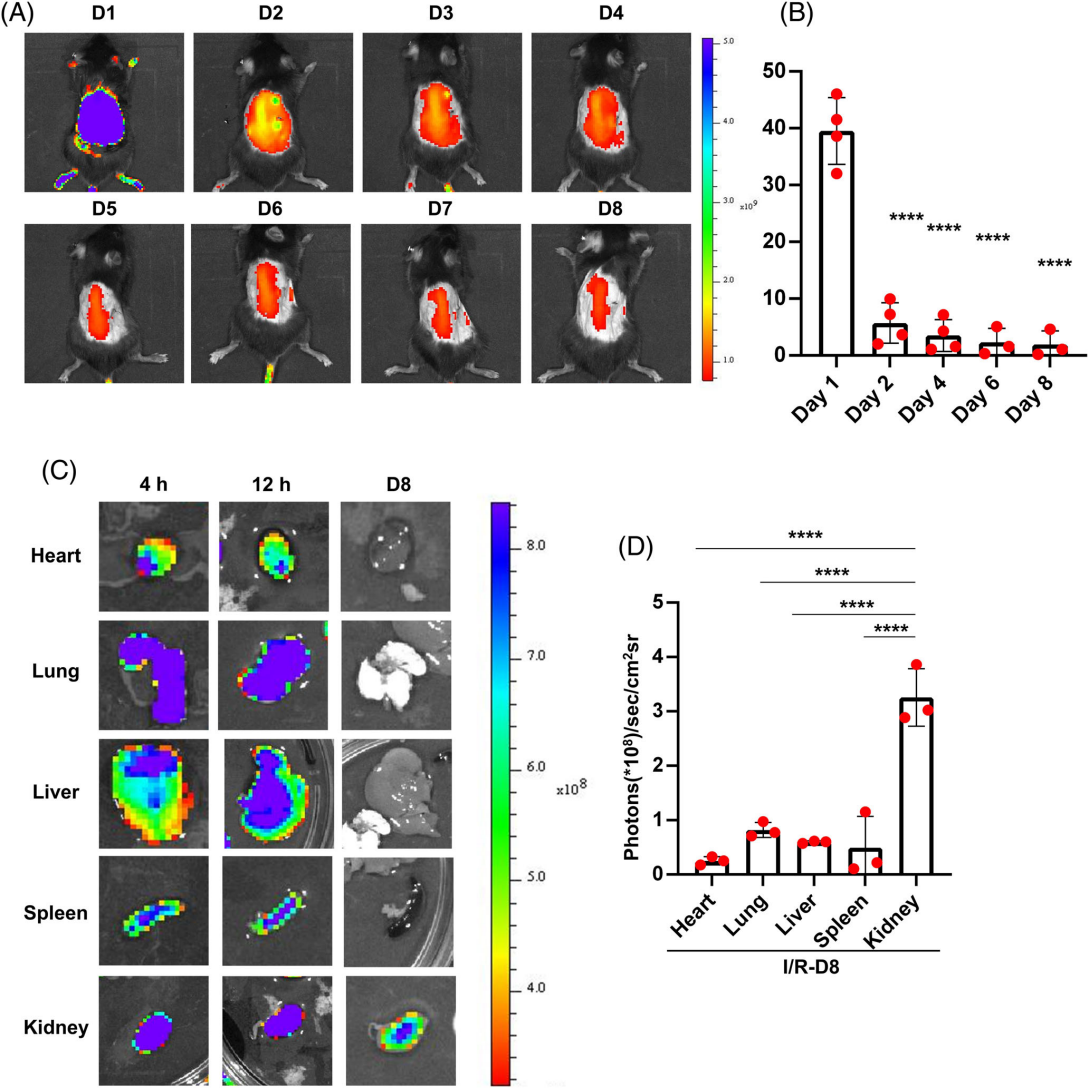
In vivo fluorescence imaging of P144 distribution. (A, B) Mice were used to generate a uIRIx model and were then injected with 1 mg/kg of the labeled P144 at D21 postsurgery. Fluorescence imaging was performed after intravenous injection of labeled P144 at indicated times. (C, D) Biodistribution of Alexa Fluor 647‐labeled P144 in different organs at indicated times after intravenous injection. Mice were sacrificed at the indicated time after P144 injection and different tissues were isolated for biodistribution evaluation. *n* = 3–4, ****p < 0.0001. uIRIx, unilateral ischemia–reperfusion injury and contralateral nephrectomy

Before the in vivo study, we labeled P144 with a near‐infrared dye, Alexa Fluor 647‐NHS ester. We injected the labeled P144 into uIRIx mice through the tail vein, and monitored the distribution of P144 using near‐infrared fluorescence imaging at different time points. We found that Alexa Fluor 647‐labeled P144 was diffused throughout the whole body after 1 D and was later enriched in the kidney, before being gradually cleared from the body (Figure 4A,B).

Mice were sacrificed at the indicated time points after the injection of P144, and different tissues were collected for further bio‐distribution evaluation. Consistent with the fluorescence imaging data, we noticed that the strongest fluorescent signal was detected in the kidney, whereas only weak signals were detected in the heart, liver, spleen, and lung at D1 (Figure 4C). We also detected the retainment of P144 in the kidney on D8 after injection, whereas only negligible signals were detected in other tissues on D8 (Figure 4D). These data suggested that P144 was enriched in the kidney in vivo.

### 3.5 P144 attenuated renal fibrosis through compromising the M2 microphage polarization and blocking TGF‐β1 signaling pathway

**FIGURE 5:**
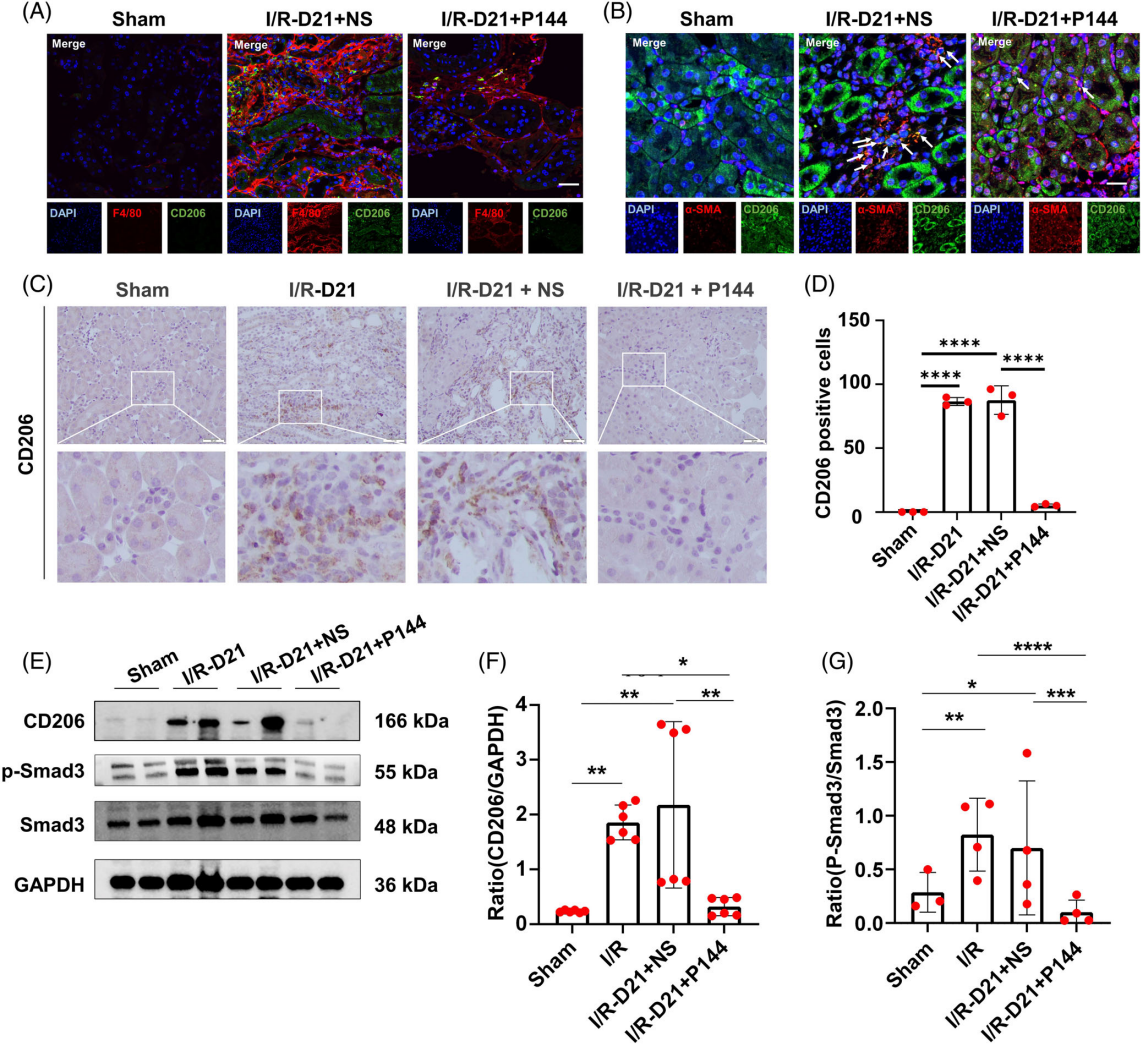
M2 microphage polarization and TGF‐β1 signaling pathway after P144 treatment. (A) CD206 positive macrophages in the kidney of uIRIx mice treated with P144 detected by immunofluorescence staining. (B) Expression of α‐SMA in CD206 positive macrophages in the kidney of uIRIx mice treated with P144 detected by immunofluorescence staining. The white arrows indicate the α‐SMA^+^ CD206^+^ macrophages. (C, D) Immunohistochemical staining of CD206 in the kidney of uIRIx mice treated with P144. (E–G) Expression of CD206, p‐Smad3, and Smad3 in the kidney of uIRIx mice treated with P144 detected by western blotting. Data are presented as the mean ± SD, *n* = 3–6. *p < 0.05, **p < 0.01, ***p < 0.001, ****p < 0.0001. Scale bar = 50 μm. TGF‐β1, transforming growth factor‐β; uIRIx, unilateral ischemia–reperfusion injury and contralateral nephrectomy

As shown in Figure 2F, macrophages predominantly infiltrated the interstitial space of the kidney after IRI. We hence further investigated whether macrophages were relevant to the therapeutic effect of P144. Staining of CD206, a surface marker of M2 macrophages, indicated that the accumulation of M2 macrophages in the fibrotic area upon IR injury was dramatically compromised by treatment with P144 (Figure 5A,C,D). Correspondingly, we found that the expression of CD206 was reduced in P144‐treated mice compared with that in untreated mice as indicated by western blotting (Figure 5E,F). As shown in Figure 5B, the expression of α‐SMA by CD206+ macrophages in the kidney of P144 treated mice was attenuated compared with untreated ones, which indicated that the transition of CD206+ macrophages to myofibroblasts might be inhibited by P144. Moreover, detection of Smad‐3 on the TGF signaling pathway showed that TGF signaling was blocked in P144‐treated kidneys (Figure 5E,G).

### 3.6 P144 attenuated the migration and polarization but not the proliferation of macrophages in vitro

**FIGURE 6:**
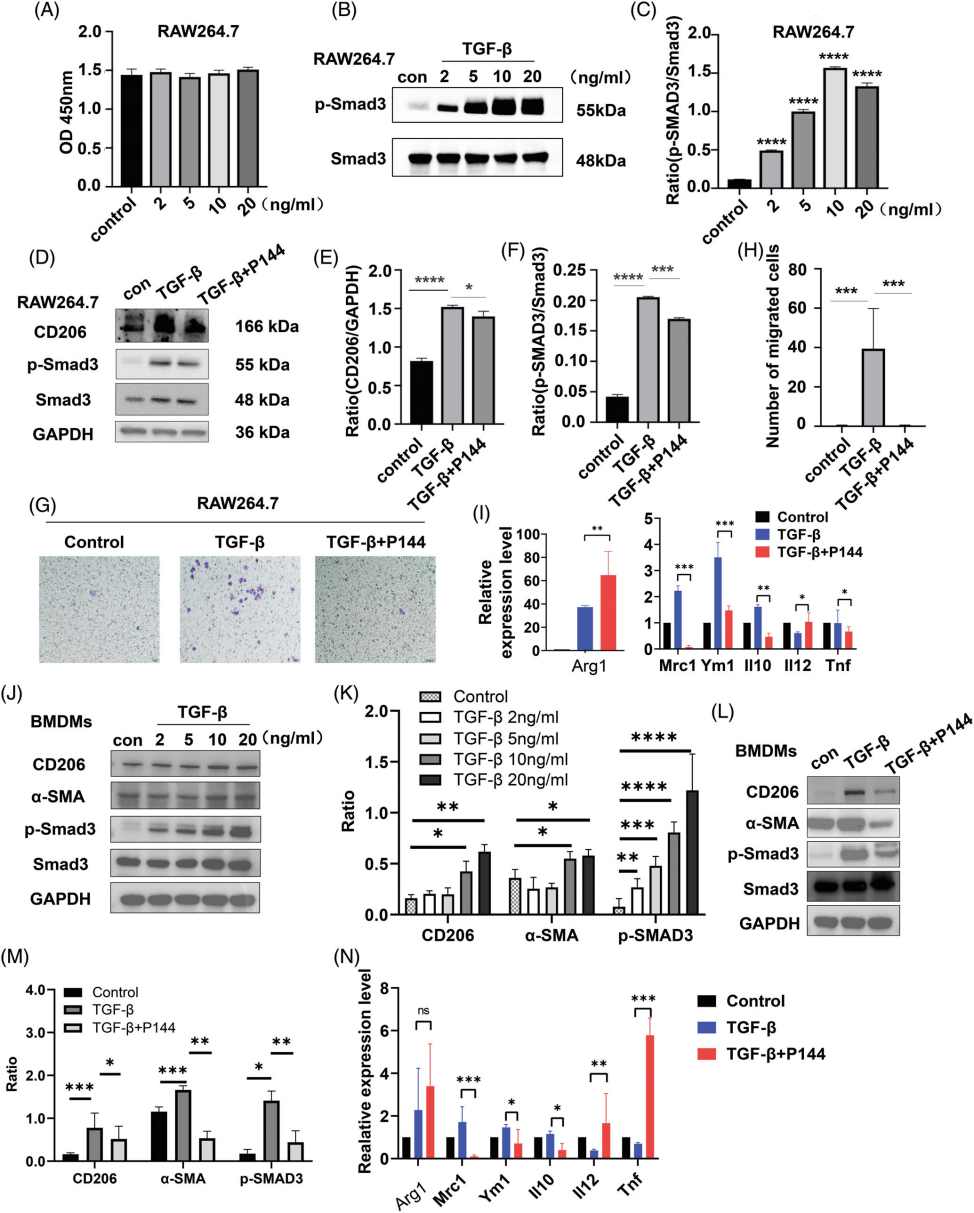
Migration and polarization of macrophages after P144 treatment in vitro. (A) The proliferation of RAW264.7 cells treated with TGF‐β1 was measured using a CCK‐8 assay. (B, C) Expression of p‐Smad3 in RAW264.7 cells treated with different doses of TGF‐β1 measured by western blotting. (D–F) Expression of CD206 and p‐Smad3 in RAW264.7 cells treated with TGF‐β1 and P144 measured by western blotting. (G, H) Migration of RAW264.7 cells treated with TGF‐β1 and P144 measured using the transwell migration assay; the number of migrated cells was quantified. (I) mRNA expression of M1 and M2 macrophage effectors in RAW264.7 cells measured by RT‐qPCR. (J, K) Expression of CD206, α‐SMA, and p‐Smad3 in BMDMs treated with TGF‐β1 measured by western blotting. (L, M) Expression of CD206, α‐SMA, and p‐Smad3 in BMDMs treated with TGF‐β1 and P144 measured by western blotting. (N) mRNA expression of M1 and M2 macrophage effectors in BMDMs measured by RT‐qPCR. Data are presented as the mean ± SD, *n* = 3, *p < 0.05, ***p < 0.001, ****p < 0.0001. BMDMs, bone marrow‐derived macrophages; TGF‐β1,transforming growth factor‐β

To confirm the effect of P144 on the phenotype and function of macrophages, we evaluated the proliferation of the RAW264.7 macrophage cell line upon treatment with TGF‐β1 and P144 using the CCK‐8 assay. We found that treatment with increasing concentrations of TGF‐β1 did not influence the viability and proliferation of macrophages (Figure 6A–C). However, TGF‐β1 induced the expression of CD206 in macrophages through the activation of Smad‐3 signaling (Figure 6D–F), and promoted the migration of macrophages, as shown in the transwell assay (Figure 6G,H). In BMDMs, TGF‐β1 treatment also increased the expression of CD206 and activated the SMAD3 signaling pathway (Figure 6J,K). Notably, treatment with P144 compromised the upregulation of the expression of CD206 in both RAW264.7 and BMDMs (Figure 6D–F,L,M), and cell migration was also reduced (Figure 6G,H). We also measured the expression of α‐SMA by BMDMs and found that after the stimulation of TGF‐β1, BMDMs upregulated the expression of α‐SMA (Figure 6J,K ), which indicated that macrophages could present myofibroblast‐like phenotype and promote the progression of renal fibrosis. Moreover, treatment with P144 reduced the expression of α‐SMA in BMDMs (Figure 6L,M), which indicated that P144 reduced the macrophage‐myofibroblast‐transition (MMT) and further ameliorated renal fibrosis.

We also tested the mRNA expression of M1 and M2 macrophage effectors in both RAW264.7 macrophage cell line and BMDMs. We found that TGF‐β1 treated macrophages upregulated the expression of Arg1, Mrc1, Ym‐1, and Il10, and downregulated the expression of Il12 and Tnf, which suggested that TGF‐β1 induced M2 polarization of macrophages. After the treatment of P144, the expression of M2 macrophage markers, such as Mrc1, Ym‐1, and Il10 was reduced, indicating that P144 attenuated the M2 polarization of macrophages induced by TGF‐β1, however, the expression of Arg1 was increased after the treatment of P144, which indicated that the P144 attenuated the M2 polarization of macrophages in an Arg‐1‐independent manner. The in vitro study showed that P144 blocked the migration and polarization of macrophages to an M2‐like phenotype induced by TGF‐β1, but had no effect on their proliferation, and further investigation is needed to uncover the underlying mechanisms.

## 4 DISCUSSION

Renal fibrosis is caused by a variety of reasons, and is characterized by the proliferation of myofibroblasts and the deposition of extracellular matrix in the renal tubule‐interstitium, which ultimately leads to the loss of renal function. Clinically, IRI‐induced AKI is the main cause of CKD. TGF‐β1 plays versatile roles in immune regulation and is known to be one of the key regulators in renal fibrosis. In particular, TGF‐β1 induces epithelial‐to‐mesenchymal transition^14^, ^15^ and drives the transition of fibroblasts to myocardial fibroblasts,^16^ which in turn produce extracellular matrix and remodel the tissue environment. The epithelial‐to‐mesenchymal transition process is also driven by canonical TGFβ1–Smad3 signaling and non‐canonical JNK signaling.^17^, ^18^

The P144 TGF‐β1 inhibitor is a functionally activated TGF‐β1 inhibitor, and in vivo experiments have shown that it effectively inhibits liver and myocardial fibrosis. Blocking TGF‐β1 in animal models of kidney disease has been shown to reduce fibroblast activation and collagen deposition.^19^, ^20^ In our study, we also found the anti‐fibrotic effect of P144 in renal IRI, which was depended on blocking the TGF‐β1 signaling pathway.

We also found that the expression of TGF‐β1 increased after D3 and was maintained until D21 post‐IRI; however, the specific role of TGF‐β1 in IRI and the subsequent renal fibrosis appeared to be time and scenario dependent. The absence of TGF‐β1 in the acute phase exacerbated ischemic renal damage,^21^ which was consistent with the aggravated fibrosis observed when IRI mice were treated with P144 at D2 post‐renal injury. This indicated that TGF‐β1 maintained epithelial integrity and promoted wound healing at the early stage of IRI. Whereas, in the late phase of IRI, TGF‐β1 signaling appeared to form a positive circuit between injured renal tube cells and activated fibroblasts and accelerated the progression of renal fibrosis.^22^ As shown in previous studies, conditional inhibition of TGF‐β1 effectively reduced IRI‐induced fibrosis.^23^, ^24^ Here, we found that P144 administered at the late phase of IRI exerted anti‐fibrotic effects. The reverse effect of P144 indicated that TGF‐β1 plays versatile roles in the repair and malfunction of renal tubule cells. The mechanism through which the progression of IRI‐induced fibrosis is regulated by TGF‐β1 both temporally and spatially remains to be explored.^16^

The role of M2 macrophages in the development and progression of renal fibrosis was also deciphered in this study. Macrophages are the dominant immune cells that infiltrate the renal interstitium after IRI. Proximal renal tubular epithelial cells are susceptible to hypoxia, which leads to programmed cell death or necrosis, triggering an inflammatory response.^25^ This was consistent with the infiltration of leukocytes cells observed on D1 and D3 post‐IRI in our study. Following the acute inflammatory stage, macrophages secrete a variety of growth factors, resulting in a repair phenotype. Studies have shown that macrophages promote renal fibrosis^26^ through a variety of mechanisms, including the production and activation of TGF‐β1. However, whether macrophages are the source of TGF‐β1 remains controversial. Huen et al.^27^ showed that macrophage‐specific TGF‐β1 deficiency had no effect on the progression of renal fibrosis in both UUO and renal IRI models. Consistently, we found that renal tubule epithelial cells, rather than macrophages, were the main source of TGF‐β1. Thus, macrophages might be downstream of TGF‐β1 in IRI‐induced renal fibrosis.

As shown in the study, the expression of α‐SMA in the kidney of P144‐treated mice was attenuated compared with untreated ones, which indicated that the transition of fibroblasts to myofibroblasts might be inhibited by P144, because the expression of α‐SMA correlates with the activation of myofibroblasts. Of cause, the direct effect of P144 on fibroblasts is important for the suppression of I/R‐induced renal fibrosis, we try to explore other mechanisms of TGF‐β1 pathway in renal I/R injury and the fibrosis, especially through innate immune cells. Macrophages are the major myeloid cells infiltrated in the renal interstitium after I/R, so we focus on the polarization and function of macrophage regulated by TGF‐b pathway, and the corresponding effect of P144 on macrophage. As shown in this study, renal fibrosis progressed post‐IRI, and was associated with an increase in the number of macrophages, especially CD206+ macrophages. CD206+ M2 macrophages have been closely related to renal fibrosis in previous studies.^28^ In addition, TGF‐β1 induced the M2 polarization of macrophages,^29^ characterized by the increased expression of CD206, whereas the addition of P144 significantly blocked the upregulation of CD206. We considered that macrophages might be one of the upstream pathways of myofibroblast activation. We found that macrophages presented myofibroblast‐like phenotype after the stimulation of TGF‐β1, manifested by increased expression of α‐SMA, and treatment with P144 reduced the expression of α‐SMA in macrophages, which indicated that P144 ameliorated renal fibrosis partially by reducing the MMT.

In summary, we analyzed the therapeutic potential of P144 in IRI‐induced renal fibrosis, and explored the underlying mechanism. In the IRI‐fibrosis model we established, the levels of both creatinine and urea nitrogen and the accumulation of extracellular matrix in the kidney were decreased after treatment with the P144 TGF‐β1 inhibitory peptide. P144 downregulated the phosphorylation of Smad3 at both the transcriptional and translational levels, and greatly dampened the infiltration of M2 macrophages in the kidney. Additionally, P144 blocked the polarization of macrophages to the M2‐like phenotype and suppressed the TGF‐β1‐induced migration of macrophages in vitro. Altogether, we found that the P144 TGF‐β1 inhibitory peptide decreased renal fibrosis through the blockade of the TGF‐β1–Smad3 signaling pathway and the modulation of macrophage polarization, suggesting its potential therapeutic use in IRI‐induced renal fibrosis.

## AUTHOR CONTRIBUTIONS

Delun Li and Qing Ouyang designed the study. Qing Ouyang, Delun Li, and Siyu Yuan wrote the manuscript. Delun Li, Jian Zhang, Chao Wang, Jiakai Chang, Yan Tong, Ran Liu, and Tian Sang performed the in vitro and in vivo experiments. Lili Li contributed to the synthesis of the TGF‐β1 peptide‐based inhibitor P144. Qing Ouyang and Jijun Li interpreted the revised data. Qing Ouyang and Xiangmei Chen supervised the study and edited the manuscript. All authors contributed to the manuscript and approved the submitted version.

## CONFLICT OF INTEREST

The author declares that there are no conflict of interest.

## Data Availability

All data and models generated and used during the study are available from the corresponding author upon reasonable request.

## References

[1] Ronco C , Bellomo R , Kellum JA . Acute kidney injury. Lancet. 2019;394:1949‐1964. doi:10.1016/S0140-6736(19)32563-2 31777389

[2] Webster AC , Nagler EV , Morton RL , Masson P . Chronic kidney disease. Lancet. 2017;389:1238‐1252. doi:10.1016/S0140-6736(16)32064-5 27887750

[3] Gu YY , Liu XS , Huang XR , Yu XQ , Lan HY . Diverse role of TGF‐beta in kidney disease. Front Cell Dev Biol. 2020;8:123. doi:10.3389/fcell.2020.00123 32258028 PMC7093020

[4] Broekema M , Harmsen MC , van Luyn MJ , et al. Bone marrow‐derived myofibroblasts contribute to the renal interstitial myofibroblast population and produce procollagen I after ischemia/reperfusion in rats. J Am Soc Nephrol. 2007;18:165‐175. doi:10.1681/ASN.2005070730 17135399

[5] Wollin L , Wex E , Pautsch A , et al. Mode of action of nintedanib in the treatment of idiopathic pulmonary fibrosis. Eur Respir J. 2015;45:1434‐1445. doi:10.1183/09031936.00174914 25745043 PMC4416110

[6] Cheng F , Shen Y , Mohanasundaram P , et al. Vimentin coordinates fibroblast proliferation and keratinocyte differentiation in wound healing via TGF‐beta‐slug signaling. Proc Natl Acad Sci U S A. 2016;113:E4320‐E4327. doi:10.1073/pnas.1519197113 27466403 PMC4968728

[7] Ezquerro IJ , Lasarte JJ , Dotor J , et al. A synthetic peptide from transforming growth factor beta type III receptor inhibits liver fibrogenesis in rats with carbon tetrachloride liver injury. Cytokine. 2003;22:12‐20. doi:10.1016/S1043-4666(03)00101-7 12946101

[8] Llopiz D , Dotor J , Casares N , et al. Peptide inhibitors of transforming growth factor‐beta enhance the efficacy of antitumor immunotherapy. Int J Cancer. 2009;125:2614‐2623. doi:10.1002/ijc.24656 19530254

[9] Dotor J , López‐Vázquez AB , Lasarte JJ , et al. Identification of peptide inhibitors of transforming growth factor beta 1 using a phage‐displayed peptide library. Cytokine. 2007;39:106‐115. doi:10.1016/j.cyto.2007.06.004 17804251

[10] Díaz‐Valdés N , Basagoiti M , Dotor J , et al. Induction of monocyte chemoattractant protein‐1 and interleukin‐10 by TGF beta 1 in melanoma enhances tumor infiltration and immunosuppression. Cancer Res. 2011;71:812‐821. doi:10.1158/0008-5472.CAN-10-2698 21159663

[11] Hermida N , López B , González A , et al. A synthetic peptide from transforming growth factor‐beta1 type III receptor prevents myocardial fibrosis in spontaneously hypertensive rats. Cardiovasc Res. 2009;81:601‐609. doi:10.1093/cvr/cvn315 19019833

[12] Qiu SS , Dotor J , Hontanilla B . Effect of P144® (anti‐TGF‐β1) in an “in vivo” human hypertrophic scar model in nude mice. PLOS ONE. 2015;10:e0144489. doi:10.1371/journal.pone.0144489 26720517 PMC4697841

[13] Ou‐Yang Q , Yan B , Li A , et al. Construction of humanized anti‐HER2 single‐chain variable fragments (husFvs) and achievement of potent tumor suppression with the reconstituted husFv‐Fdt‐tBid immunoapoptotin. Biomaterials. 2018;178:170‐182. doi:10.1016/j.biomaterials.2018.06.016 29935385

[14] Ng YY , Fan JM , Mu W , et al. Glomerular epithelial‐myofibroblast transdifferentiation in the evolution of glomerular crescent formation. Nephrol Dial Transplant. 1999;14:2860‐2872. doi:10.1093/ndt/14.12.2860 10570089

[15] Piera‐Velazquez S , Li Z , Jimenez SA . Role of endothelial‐mesenchymal transition (EndoMT) in the pathogenesis of fibrotic disorders. Am J Pathol. 2011;179:1074‐1080. doi:10.1016/j.ajpath.2011.06.001 21763673 PMC3157273

[16] Hong KM , Belperio JA , Keane MP , Burdick MD , Strieter RM . Differentiation of human circulating fibrocytes as mediated by transforming growth factor‐beta and peroxisome proliferator‐activated receptor gamma. J Biol Chem. 2007;282:22910‐22920. doi:10.1074/jbc.M703597200 17556364

[17] Yang Q , Ren GL , Wei B , et al. Conditional knockout of TGF‐β1RII/Smad2 signals protects against acute renal injury by alleviating cell necroptosis, apoptosis and inflammation. Theranostics. 2019;9:8277‐8293. doi:10.7150/thno.35686 31754396 PMC6857044

[18] Meng XM , Nikolic‐Paterson DJ , Lan HY . TGF‐β1: the master regulator of fibrosis. Nat Rev Nephrol. 2016;12:325‐338. doi:10.1038/nrneph.2016.48 27108839

[19] De Silva R , Reddel RR . Similar simian virus 40‐induced immortalization frequency of fibroblasts and epithelial cells from human large airways. Cell Mol Biol Res. 1993;39:101‐110. 8220580

[20] Baltanás A , Miguel‐Carrasco JL , San José G , et al. A synthetic peptide from transforming growth factor‐β₁ type III receptor inhibits NADPH oxidase and prevents oxidative stress in the kidney of spontaneously hypertensive rats. Antioxid Redox Signal. 2013;19(14):1607‐1618. doi:10.1089/ars.2012.4653 23350688

[21] Guan Q , Nguan CY , Du C . Expression of transforming growth factor‐beta1 limits renal ischemia‐reperfusion injury. Transplantation. 2010;89:1320‐1327. doi:10.1097/TP.0b013e3181d8e9dc 20458271

[22] Luo C , Zhou S , Zhou Z , et al. Wnt9a promotes renal fibrosis by accelerating cellular senescence in tubular epithelial cells. J Am Soc Nephrol. 2018;29:1238‐1256. doi:10.1681/ASN.2017050574 29440280 PMC5875944

[23] Qiao X , Rao P , Zhang Y , et al. Redirecting TGF‐beta signaling through the beta‐catenin/foxo complex prevents kidney fibrosis. J Am Soc Nephrol. 2018;29:557‐570. doi:10.1681/ASN.2016121362 29180394 PMC5791062

[24] Yang K , Li W , Bai T , et al. Mindin deficiency alleviates renal fibrosis through inhibiting NF‐κB and TGF‐β1/Smad pathways. J Cell Mol Med. 2020;24:5740‐5750. doi:10.1111/jcmm.15236 32253812 PMC7214143

[25] Tammaro A , Kers J , Scantlebery AML , Florquin S . Metabolic flexibility and innate immunity in renal ischemia reperfusion injury: the fine balance between adaptive repair and tissue degeneration. Front Immunol. 2020;11:1346. doi:10.3389/fimmu.2020.01346 32733450 PMC7358591

[26] Nikolic‐Paterson DJ , Wang S , Lan HY . Macrophages promote renal fibrosis through direct and indirect mechanisms. Kidney Int Suppl. 2014;4:34‐38. doi:10.1038/kisup.2014.7 PMC4536961 26312148

[27] Huen SC , Moeckel GW , Cantley LG . Macrophage‐specific deletion of transforming growth factor‐beta1 does not prevent renal fibrosis after severe ischemia‐reperfusion or obstructive injury. Am J Physiol Renal Physiol. 2013;305:F477‐F484. doi:10.1152/ajprenal.00624.2012 23761668 PMC3891258

[28] Tang PM , Nikolic‐Paterson DJ , Lan HY . Macrophages: versatile players in renal inflammation and fibrosis. Nat Rev Nephrol. 2019;15:144‐158. doi:10.1038/s41581-019-0110-2 30692665

[29] Kim MG , Kim SC , Ko YS , Lee HY , Jo SK , Cho W . The role of M2 macrophages in the progression of chronic kidney disease following acute kidney injury. PLOS ONE. 2015;10:e0143961. doi:10.1371/journal.pone.0143961 26630505 PMC4667939

